# Induced resistance in groundnut by jasmonic acid and salicylic acid through alteration of trichome density and oviposition by *Helicoverpa armigera* (Lepidoptera: Noctuidae)

**DOI:** 10.1093/aobpla/plt053

**Published:** 2013-11-26

**Authors:** Abdul Rashid War, Barkat Hussain, Hari C. Sharma

**Affiliations:** 1 International Crops Research Institute for the Semi-Arid Tropics (ICRISAT), Patancheru, Andhra Pradesh 502 324, India; 2 Division of Entomology, SKUAST-K, Srinagar, India

**Keywords:** Groundnut, induced resistance, oviposition, phytohormones, trichomes.

## Abstract

Jasmonic acid (JA) and salicylic acid (SA) are important phytohormones involved in plant resistance against insect herbivory and pathogen infection. Application of JA and SA induces several defensive traits in plants. Here we investigated the effect of JA and SA on trichome density in five groundnut genotypes [ICGV 86699, ICGV 86031, ICG 2271, ICG 1697 (resistant) and JL 24 (susceptible)]. The effect of JA- and SA-induced resistance on the oviposition behaviour of *Helicoverpa armigera* on different groundnut genotypes was also studied. Pre-treatment with JA increased numbers of trichomes in the insect-resistant genotypes, ICGV 86699, ICGV 86031, ICG 2271, and ICG 1697. The induction was greater at 10 days after treatment. Jasmonic acid- and SA-treated plants showed a substantial effect on the oviposition behaviour of *H. armigera.* Jasmonic acid application and herbivory reduced the number of eggs laid by *H. armigera* in all the groundnut genotypes tested. However, a greater reduction was recorded on plants pre-treated with JA. More egg laying was recorded in JL 24 in all the treatments as compared to the insect-resistant genotypes. These results suggested that pre-treatment with JA increased trichome density in groundnut plants, which conferred antixenosis for oviposition by *H. armigera*.

## Abstract (precis)

Jasmonic acid (JA) and salicylic acid (SA) play an important role in activating plant defensive responses against insect pests. In these experiments, trichome density increased in groundnut plants by the pretreatment of JA and SA; however, JA induced significantly greater number of trichomes than SA. Moreover, JA activated antixenosis for oviposition by *H. armigera*. Insect resistant genotypes (ICGV 86699, ICGV 86031, ICG 2271 and ICG 1697) showed stronger response than the JL 24 (susceptible check). These results show that pre-treatment with JA not only resulted in greater trichome density in groundnut plants, but also conferred antixenosis for oviposition by *H. armigera*.

## Introduction

Plants face a great threat from insects, which are the main constraints in crop production. However, they have developed various strategies to avoid and/or reduce insect damage. These include morphological, physiological and biochemical features. The structural defence forms the first line of defence against insects, and comprises the morphological and anatomical traits that are advantageous to plants, which deter the insect herbivores (Hanley *et al.* 2007; Agrawal *et al.* 2009). Among them, trichomes are the most important structural features of plant defence against insect pests (Sharma *et al.* 2009; He *et al.* 2011). These are hairy structures present on the stem, leaves and fruits. They can be straight, spiral, stellate or hooked (Agrawal 1999; Hanley *et al.* 2007). Broadly, trichomes are classified as glandular and non-glandular. Non-glandular trichomes are involved in physical defence of the plants, while glandular trichomes defend plants physically as well as chemically. Glandular trichomes secrete defensive secondary metabolites, including flavonoids, terpenoids and alkaloids, which are toxic to insect pests (Handley *et al.* 2005). Trichomes and their exudates influence both larval feeding and oviposition by insects (Handley *et al.* 2005). Induction of trichomes in plants in response to herbivory is considered an important defensive strategy to minimize subsequent damage by the herbivores (Agrawal 1999; Traw and Dawson 2002). Alteration of trichome density in plants occurs within days or weeks after insect damage (Agrawal 1999; Dalin and Björkman 2003).

Host plant selection for oviposition is crucial as the suitability of the host plant will determine the survival and development of the progeny. Surface chemicals, plant volatiles and trichomes have a major influence on the oviposition behaviour of insects (Hilker *et al.* 2002; Chamarthi *et al.* 2011). Antixenosis for oviposition is the most important plant defence against insect herbivory. Various physical and chemical cues are utilized by the female moths to select a suitable host plant for oviposition. Plants respond to insect oviposition through direct and indirect defences, which aim to get rid of the insect eggs and/or to kill them, thus avoiding the damage by the larvae that would hatch from them (Hilker and Meiners 2010). Plants in response to oviposition produce neoplasm at the egg deposition site, which elevates the eggs and drops them down (Doss *et al.* 2000) and also produce ovicidal compounds that kill the eggs (Seino *et al.* 1996; Yamasaki *et al.* 2003). In addition, oviposition induces necrotic tissue formation at the oviposition site by the hypersensitive response of plant tissues, which detaches the eggs (Petzold-Maxwell *et al.* 2011).

Jasmonic acid (JA) and salicylic acid (SA) are the important phytohormones involved in plant defence against insect herbivory (Stotz *et al.* 2002; Traw and Bergelson 2003; Bruinsma *et al.* 2007; Zhao *et al.* 2009). They induce toxic secondary metabolites and antinutritive compounds in plants, which reduce larval growth and development and deter adult moths from oviposition (Bruinsma *et al.* 2007). Octadecanoid and phenylpropanoid pathways mediated by JA and SA, respectively, release a number of intermediary compounds. Some of these compounds have an antibiotic effect on insect pests, while others show an antixenotic effect for oviposition (van Poecke and Dicke 2002; Bruinsma *et al.* 2007). It has been reported that JA and SA pathways act antagonistically (Traw and Bergelson 2003). Plants treated with JA received a lower number of eggs from *Pieris rapae* and *Pieris brassicae* as compared with the untreated control plants (Bruinsma *et al.* 2007). Furthermore, a higher concentration of JA has been found more in eggs of various lepidopteran insects than in plant tissues or larval diet (Hilker and Meiners 2010).

Groundnut plants have a good potential for induced resistance against insect pests (War *et al.* 2011). Exogenous application of JA and SA induces various plant defensive traits in groundnut, which confer resistance to insect pests (War *et al.* 2011; A. R. War *et al.,* unpubl. data). However, there are no reports on the plant morphology based induced resistance by phytohormones in groundnut. To test the hypothesis that JA- and SA-induced resistance against *Helicoverpa armigera* in groundnut could be due to the alteration in trichome production in the host plant and the altered oviposition behaviour of the target pest, the effects of JA and SA on trichome production in groundnut plants and on the oviposition behaviour of *H. armigera* were studied.

## Methods

### Groundnut plants

Groundnut plants (*Arachis hypogaea*) were raised under greenhouse conditions at the International Crops Research Institute for the Semi-Arid Tropics (ICRISAT), Patancheru, Andhra Pradesh, India. The genotypes were ICGV 86699, ICGV 86031, ICG 2271, ICG 1697 (with moderate to high levels of resistance to insects) and JL 24 (susceptible check) (Sharma *et al.* 2003). Genotypes were selected on the basis of their response to insect infestation and/or JA and SA application (War *et al.* 2011; A. R. War *et al.,* unpubl. data). Plants were grown in plastic pots (30 cm diameter and 39 cm deep) filled with soil, sand and farmyard manure (2 : 1 : 1). Two seedlings were retained in each pot. Desert coolers were used to maintain the temperature at 28 ± 5 °C and the relative humidity (RH) at 65 ± 5 % in the greenhouse.

### Insect infestation

*Helicoverpa armigera* neonates were obtained from the stock culture maintained on a chickpea-based semi-synthetic diet under laboratory conditions (26 ± 1 °C; 11 ± 0.5 h photoperiod and 75 ± 5 % RH) from the insect rearing laboratory at ICRISAT. Ten newly hatched larvae were gently placed on each 20-day-old plant by using a camel hair brush.

### Treatments

Plants were treated with JA and SA (Sigma Aldrich, USA) to study their role in induced resistance in groundnut against *H. armigera*. The JA and SA were sprayed until runoff at a concentration of 1 mM as standardized earlier for groundnut (War *et al.* 2011; A. R. War *et al.,* unpubl. data).

#### Effect of JA, SA and insect infestation on trichome density of groundnut plants

There were four treatments for each genotype. Treatment I: the plants were pre-treated with JA (1 mM) for 24 h and then infested with *H. armigera* (PJA + HA); Treatment II: the plants were pre-treated with SA for 24 h and then infested with *H. armigera* (PSA + HA); Treatment III: the plants were pre-infested with *H. armigera* (PHI) for 24 h; and Treatment IV: the unsprayed and uninfested plants were maintained as a control.

After 5 and 10 days of treatment (DAT), newly expanded tetrafoliates were collected from each plant and used to record the trichome density. The tetrafoliates from the treated and untreated plants were immersed in water and incubated at 70 °C for 2–4 min. The samples were cleared in 90 % ethanol for 1 day and transferred to ethanol : acetic acid (2 : 3 ratio) for 24 h. The leaf samples were stored in 90 % lactic acid solution. To record the trichome density, the leaves were examined at a magnification of ×100 under a stereomicroscope (Olympus 598472, Japan). The trichome count was taken randomly at five places in each leaf on the adaxial surface and the average trichome density was expressed as the number of trichomes per square millimetre.

#### Effect of JA, SA and insect infestation on oviposition behaviour of *H. armigera*

Two plants were retained in each pot after 10 days of emergence, and 20-day-old plants were used for the experiment. One plant in each pot was covered with a plastic cage (11 cm diameter, 26 cm in height). Newly emerged *H. armigera* adults were used for oviposition. Plants in each genotype were divided into six groups. Group I: plants pre-treated with 1 mM JA for 1 day and one pair (one male and one female) of *H. armigera* released inside the cage (PJA + HA); Group II: plants pre-treated with 1 mM SA for 1 day prior to the release of one pair of *H. armigera* adults (PSA + HA); Group III: plants pre-infested for 1 day with three third-instar larvae of *H. armigera* and one pair of *H. armigera* adults released inside the cage (PHI + HA); Group IV: plants sprayed with 1 mM JA and one pair of *H. armigera* adults released at the same time (JA + HA); Group V: plants sprayed with 1 mM SA and one pair of *H. armigera* adults released simultaneously (SA + HA); and Group VI: only a pair of *H. armigera* adults released on untreated plants (HA). The adults were provided with 10 % sucrose solution and kept inside the cage for 6 days. After 6 days, the adults were removed from the plants and the numbers of eggs laid on the plants were recorded. Eggs on the walls and lid of the jar were not taken into consideration. The neonates on some plants were also counted as eggs.

### Statistical analysis

The data were subjected to analysis of variance (ANOVA) using SPSS (15.1). Tukey's/multiple comparison tests were used to separate the means when the treatment effects were statistically significant (*P* ≤ 0.05). Correlation analysis was performed to see the association between trichome density and oviposition (*P* ≤ 0.05).

## Results

### Effect of JA, SA and insect infestation on trichome density

**Figure 1.**
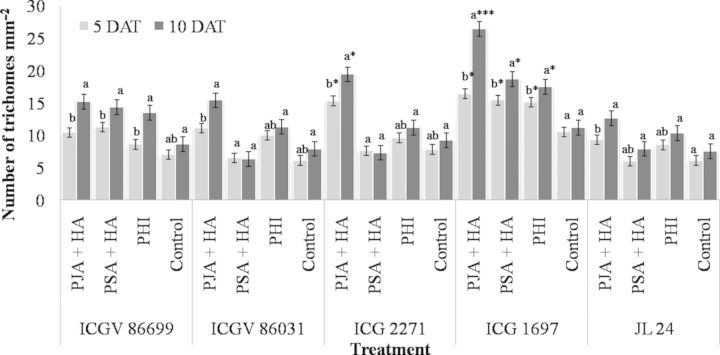
Number of trichomes (per square millimetre) on groundnut leaves pre-treated with JA and SA and infested with insects. Scale bars (mean ± SEM) of the same colour with similar letters are significantly different at *P* ≤ 0.05; asterisks indicate a significant difference in trichome number across the genotypes with ***P ≤ 0.001, **P ≤ 0.01 and *P ≤ 0.05. PJA, pre-treatment with JA and infested with *H. armigera*; PSA, pre-treatment with SA and infested with *H. armigera*; PHA, pre-infested with *H. armigera*; control, untreated and uninfested plants.

A change in trichome density was observed in plants at 5 and 10 DAT with JA, SA and *H. armigera* infestation (Fig. 1). The PJA + HA-treated plants of ICG 1697 had significantly greater number of trichomes at 10 DAT (*F*_(3,11)_ = 34.5, *P* < 0.01) as compared with PSA + HA, PHI and untreated plants. There were no significant differences in trichome numbers between PJA + HA-, PSA + HA- and PHI-treated and untreated plants at 5 DAT in ICGV 86699 (*P* > 0.05). However, at 10 DAT, a significant increase in trichome count was observed in PJA + HA- and PSA + HA-treated, and PHI plants (*F*_(3,11)_ = 21.4, *P* < 0.01) as compared with the untreated plants. In ICGV 86031 and ICG 2271, PJA + HA-treated plants showed a significant increase in trichome density both at 5 DAT (*F*_(3,11)_ = 14.5 and 27.9, respectively, *P* < 0.05) and at 10 DAT (*F*_(3,11)_ = 12.4 and 10.7, respectively, *P* < 0.05) than PSA + HA and PHI and untreated control plants. Across the genotypes at 5 DAT, ICG 2271 and ICG 1697 plants treated with PJA + HA exhibited a significantly higher trichome density (*F*_(4,14)_ = 36.9, *P* < 0.01) than ICGV 86699, ICGV 86031 and JL 24, while at 10 DAT, the PJA + HA-treated plants of ICG 1697 exhibited significantly greater number of trichomes (*F*_(4,14)_ = 49.8, *P* < 0.001) than ICGV 86699, ICGV 86031, ICG 2271 and JL 24. The PSA + HA- and PHI-treated plants of ICG 1697 showed greater trichome density at 5 DAT (*F*_(4,14)_ = 10.3 and 7.8, respectively, *P* < 0.05) and 10 DAT (*F*_(4,11)_ = 29.7 and 15.4, respectively, *P* < 0.05) than ICGV 86699, ICGV 86031, ICG 2271 and JL 24. Constitutive levels of trichomes were greater in ICG 1697 (*F*_(4,14)_ = 12.6, *P* < 0.05) as compared with the rest of the genotypes tested.

### Effect of JA, SA and insect infestation on the oviposition behaviour of *H. armigera*

The susceptible check, JL 24, was preferred for oviposition by *H. armigera* females in all the treatments as compared with ICGV 86699, ICGV 86031, ICG 2271 and ICG 1697 (Table 1). However, the number of eggs laid differed across the treatments. Among the treatments, the PJA + HA-, PHI + HA- and JA + HA-treated plants were less preferred for oviposition across genotypes (*F*_(5,17)_ = 64.3, 33.2, 36.5, 28.7 and 49.6 for ICGV 86699, ICGV 86031, ICG 2271, ICG 1697 and JL 24, respectively, *P* < 0.01) than the PSA + HA-, SA + HA- and HA-treated plants. Among the resistant genotypes, ICG 1697 plants were least preferred for egg laying in PSA + HA- and HA-treated plants (*F*_(4,14)_ = 29.6 and 16.1, respectively, *P* < 0.01) as compared with ICGV 86699, ICGV 86031, ICG 2271 and JL 24. Plants of ICGV 86699 and ICG 1697 treated with PJA + HA, PHI + HA and JA + HA were less preferred for egg laying (*F*_(4,14)_ = 32.4, 24.5 and 19.8, respectively, *P* < 0.01) as compared with ICGV 86031, ICG 2271 and JL 24. Across the genotypes, a significantly greater number of eggs was laid on JL 24 in all the treatments (*P* < 0.01). A significant and negative correlation was observed between trichome counts and number of eggs laid in treated plants: PJA + HA (*r* = −0.67), PSA + HA (*r* = −0.5) and PHI (*r* = −0.67).

**Table 1.** Eggs laid by *H. armigera* on groundnut plants treated with JA and SA. Values (mean ± SD) within a column with the same superscript lower case letters are not significantly different (*P* ≤ 0.05). Values within a row with the same superscript upper case letters are not significantly different (*P* ≤ 0.05). PJA + HA, pre-treatment with JA for 1 day and an adult pair of *H. armigera* released; PSA + HA, pre-treatment with SA for 1 day and an adult pair of *H. armigera* released; PHI + HA, pre-infested with *H. armigera* for 1 day and an adult pair of *H. armigera* released; JA + HA, JA sprayed + an adult pair of *H. armigera* released; SA + HA, SA sprayed + an adult pair of *H. armigera* released; HA, an adult pair of *H. armigera* released

Genotype	Treatment					
	PJA + HA	PSA + HA	PHI + HA	JA + HA	SA + HA	HA
ICGV 86699	50.4 ± 3.5^cB^	79.9 ± 1.8^bB^	69.7 ± 3.8^bcB^	66.5 ± 2.6^bcB^	94.8 ± 5.7^bA^	103.5 ± 5.4^bA^
ICGV 86031	65.7 ± 2.3^bB^	82.5 ± 2.6^bB^	65.5 ± 3.5^bB^	72.0 ± 4.4^bB^	89.3 ± 4.9^bB^	131.2 ± 6.9^bA^
ICG 2271	69.0 ± 5.9^bB^	85.9 ± 4.3^bB^	73.8 ± 2.7^bB^	79.5 ± 3.4^bB^	92.8 ± 4.5^bB^	137.1 ± 3.4^bA^
ICG 1697	45.4 ± 2.5^cB^	63.4 ± 4.8^cB^	54.3 ± 4.5^cB^	57.7 ± 3.7^cB^	89.5 ± 3.8^bA^	98.8 ± 5.7^cA^
JL 24	111.5 ± 3.3^aD^	144.0 ± 5.4^aC^	119 ± 3.7^aCD^	126.9 ± 5.6^aC^	174.0 ± 5.2^aB^	231.6 ± 6.5^aA^

## Discussion

Plants respond to herbivory not only through biochemical mechanisms, but also through the induction of morphological features such as trichome density in subsequent plant growth (Traw and Dawson 2002). The present study revealed an increase in the number of trichomes in groundnut plants in response to infestation with *H. armigera* and JA and SA application. Insect-infested plants pre-treated and/or simultaneously treated with JA and SA had higher numbers of trichomes than the untreated control plants. Plants of ICGV 86031 and ICG 2271 pre-treated with JA responded more strongly in terms of induction of trichomes at 5 DAT than the rest of the treatments. However, ICG 1697 showed a greater number of trichomes in PJA + HA-, PSA + HA- and PHI-treated plants than the untreated control plants. ICG 1697 had significantly greater numbers of trichomes than the rest of the genotypes at 10 DAT. This increase in trichome density in response to insect damage was observed in leaves that appeared subsequent to insect attack and/or elicitor treatment (Agrawal *et al.* 2009). Antagonistic interaction has been observed between JA and SA pathways and a decrease in trichome production in *Arabidopsis* by SA treatment (Traw and Bergelson 2003). However, we did not find any such interaction between JA and SA in groundnut in terms of trichome production. More importantly, plants pre-treated with SA had a trichome density on a par with those of insect-infested plants in ICGV 86699, ICG 1697 and JL 24 at 10 DAT. The alteration in expression of genes responsible for trichome production in response to external stimuli by insect herbivory and/or elicitor application leads to the differences in trichome density in plants (Kivimäki *et al.* 2007). Induction of trichome density in response to insect infestation and/or elicitor application will have a substantial effect in controlling insect herbivory. The dense covering of trichomes affects the herbivores mechanically, and interferes with the movement of insects and other arthropods on the plant surface, thereby reducing their access to the leaf epidermis. Removal of trichomes makes leaves more susceptible to insect attack (Fordyce and Agrawal 2001; Agrawal *et al.* 2009). There is considerable evidence to suggest an increase in density of trichomes in plants in response to herbivory and/or elicitor application (Agrawal 1999; Traw and Dawson 2002; Bjorkman and Ahrne 2005). However, infestation by *P. rapae* increased the density of trichomes in black mustard (Traw and Dawson 2002). Jasmonic acid and methyl jasmonate application resulted in a greater number of trichomes in *Arabidopsis* and tomato (Traw and Bergelson 2003; Boughton *et al.* 2005).

Insect oviposition is the first encounter between most of the insect pests and host plants, and oviposition preference or non-preference is the most important step to determine plant resistance and/or susceptibility to the insect pests. Successful oviposition will result in successful emergence of the larvae and greater infestation. So plants have evolved various defensive tactics to avoid oviposition by insect pests. Any effect on the oviposition behaviour of insects will have an effect on the level of infestation. Jasmonic acid and SA application and herbivory reduced the number of eggs laid by *H. armigera* in all the groundnut genotypes tested as compared with the control plants; however, a stronger effect was observed in plants pre-treated with JA as compared with the rest of the treatments. Reduced oviposition on plants treated with JA or SA or damaged by insects could be attributed to changes in volatile compounds and changes in trichome density. There was greater oviposition by *H. armigera* on JL 24 than the insect-resistant genotypes. Bruinsma *et al.* (2007) reported that *Brassica oleracea* plants treated with JA showed a reduction in oviposition by *P. rapae* and *P. brassicae* females. Methyl salicylate (MeSA) inhibits oviposition by the cabbage moth *Mamestra brassicae* (Ulland *et al.* 2008), suggesting that MeSA can also be detected by the attacking herbivores. Infested cabbage and cotton plants have been reported to be less preferred by the cabbage looper, *Trichoplusia ni* adults for oviposition as compared with the undamaged plants (Landolt 1993).

## Conclusions

In conclusion, pre-treatment with JA increased the trichome density in groundnut plants at 10 DAT as compared with plants treated with SA. In addition, reduced egg laying by *H. armigera* was recorded on plants treated with JA. Overall, insect-resistant groundnut genotypes showed a stronger response to JA application than the susceptible check, JL 24. However, the induction of trichome production in plants in response to application of phytohormones/insect infestation should be considered in conjunction with their effects on tritrophic interactions, and fitness costs to the plant. There is a need for an in-depth understanding of such interactions at the genetic and molecular levels in order to exploit them for pest management.

## Contributions by the Authors

A.R.W. and H.C.S. planned the studies. A.R.W. and B.H. carried out the experiments. A.R.W. and H.C.S. analysed the resulting data.

## Conflicts of Interest Statement

None declared.
